# Topological Interference Management for *K*-User Downlink Massive MIMO Relay Network Channel

**DOI:** 10.3390/s17081896

**Published:** 2017-08-17

**Authors:** Poongundran Selvaprabhu, Sunil Chinnadurai, Jun Li, Moon Ho Lee

**Affiliations:** 1 Department of Electronics and Information Engineering, Chonbuk National University, Jeonju 561-756, Korea; poongundran@jbnu.ac.kr (P.S.); sunilkcsss@jbnu.ac.kr (S.C.); 2 School of Mechanical and Electrical Engineering, Guangzhou University, Guangzhou 510006, China; lijun52018@gzhu.edu.cn

**Keywords:** K-user MIMO relay channel, minimum mean square error (MMSE), interference alignment, limited CSIR knowledge

## Abstract

In this paper, we study the emergence of topological interference alignment and the characterizing features of a multi-user broadcast interference relay channel. We propose an alternative transmission strategy named the relay space-time interference alignment (R-STIA) technique, in which a K -user multiple-input-multiple-output (MIMO) interference channel has massive antennas at the transmitter and relay. Severe interference from unknown transmitters affects the downlink relay network channel and degrades the system performance. An additional (unintended) receiver is introduced in the proposed R-STIA technique to overcome the above problem, since it has the ability to decode the desired signals for the intended receiver by considering cooperation between the receivers. The additional receiver also helps in recovering and reconstructing the interference signals with limited channel state information at the relay (CSIR). The Alamouti space-time transmission technique and minimum mean square error (MMSE) linear precoder are also used in the proposed scheme to detect the presence of interference signals. Numerical results show that the proposed R-STIA technique achieves a better performance in terms of the bit error rate (BER) and sum-rate compared to the existing broadcast channel schemes.

## 1. Introduction

As the size of a wireless network increases, interference alignment becomes more complex and challenging. Hence, extensive research is being done for enlarging multiple-input-multiple-output (MIMO) communication strategies to achieve an optimal solution and to reduce the complexity of the interference channels. In particular, K -user MIMO broadcast channels (BC) with a shared relay and channel state information (CSI) are essential for enhancing the performance of a wireless broadcast channel network. It has been demonstrated in [1] that the channel state information at the transmitter (CSIT) is essential for optimizing the wireless-broadcast-channel performance. Considerable study on the design of the beamforming matrix and multiuser interference signals using the space-time interference alignment (STIA) technique is available. In [2], the authors discuss about the fully-associated interference networks, where a relay equipped with several antennas forwards the received signals instantly to the desired receivers. Precisely, the outdated CSI from all the transmitters sends the estimated CSI back to the relay with a handling delay.

Recently, the authors in [3,4] have provided a more distinguished analysis on topology interference management performance with a message passing (TIM-MP) technique for partially connected BC, where the transmitter has access only to the topological information and each receiver can pass its decoded message to another receiver. Linear minimum mean square error (MMSE) pre-equalization has been employed in [5] to precode the transmitted signal information using the Alamouti code with a precoded spatial-multiplexing technique that assists in retrieving or decoding the interference signals on the destination-side. The MMSE linear precoder achieves better error performance than the zero forcing-linear precoding method (ZL-LP) due to the presence of a large number of users and antennas at the base stations (BS) [6,7]. In order to construct a more structured and effective solution for reconstructing the interference signals, the authors in [8,9,10] introduces the retrospective interference alignment, linear precoder design and spatial multiplexing techniques, which have the ability to recover using outdated CSIT knowledge. A comparison between the downlink performance of the cell edge users and the quasi-orthogonal space-time code for MIMO systems is beneficial [11,12]. However, the authors have evaluated the performance without an inter-cell interference coordination scheme, using cooperative base stations. Moreover, the authors in [13,14] have deliberated on the topological interference alignment for MIMO relay broadcast channels under a span interference alignment method, where the desired signal information is reconstructed without appropriate CSIT knowledge.

The common assumption based on the aforementioned works is that they depend on a feedback protocol (or) delayed CSIT knowledge. The author in [15] have extended the analysis with multiuser MIMO user selection and reconfigurable antennas, for switching at the receiver-side. In [16], the author presents multiple relays or a multi-way relaying system with global CSI knowledge. In the above-mentioned schemes, the complexity of the recovered signal or the decomposed signal strength is considerably higher on the receiver-side. It is observed that increasing the number of users and relays increases the interference. In this paper, we propose a new scheme to ratify the difficulties in reconstructing a linear combination of the desired and undesired symbols, using the distributed nature of information and the delayed channel state information at the relay (CSIR) knowledge. The significant feature of this study is to reconstruct the interference signals in the MIMO interference channel from an unknown receiver with imperfect CSIR knowledge. However, this work differs from [1,2] where the authors proposed an interference alignment scheme for the K -user multiple-input-single-output (MISO) interference channel with periodic and imperfect channel knowledge, respectively. The space-time relay transmission technique assists in increasing the recovered signal strength by incorporating two unknown transmitter and relay signals efficiently. The retrieval of the desired signal information is evaluated by the combination of two unknown co-interference signals from the transmitter and the relay facilitates the reconstruction of the interference signals from the unintended (additional) receiver by considering the cooperation between the receivers. If the distance between the intended and unintended receiver is too large, the cooperation between the receivers will be affected drastically, which, in turn, will degrade the sum rate and the bit error rate (BER) performance of the proposed system.

### 1.1. Summary of Contribution

The main contributions of this paper are summarized as follows:

- We consider a three-user MIMO interference channel with a shared relay, where no more than two transmitters can be active over the same time slot. At the receiver-end, the interference signals from an unknown transmitter affect the performance of the downlink relay network channel considerably.
- The introduction of an additional (unintended) receiver incorporates one relay signal and two unknown receiver signals received from the two active transmitters aid to improving the recovered signal strength drastically at the intended receiver-end.
- The proposed R-STIA technique assists the interference signals to align in both the time and space domains under limited CSIR knowledge by using the R-STIA transmission technique. The proposed technique also recovers and reconstructs the transmitter and relay signals at the unintended receiver-end by considering the cooperation between the receivers.
- The motivation behind the proposed scheme is to reduce the transmitter power consumption largely by not activating more than two transmitters over the same time slot. The proposed scheme can also reduce the decoding complexity due to the presence of the additional receiver and less interference misalignment at the intended receiver-end.
- The Alamouti space-time transmission technique and the MMSE linear precoder are employed at the transmitters and relay to detect the presence of interference signals at the unintended receiver, which, in turn, aid in improving the BER performance of the proposed scheme.
- Numerical results confirm that the proposed R-STIA technique significantly improves the sum-rate and BER performance compared to the existing broadcast channel schemes [17,18].

### 1.2. Organization

The rest of this paper is organized as follows. Section 2 introduces the channel model and the formula definitions for the K -user MIMO interference channels. In Section 3, the proposed scheme for a three-user MIMO relay-aided broadcast channel is illustrated. Section 4 presents the precoded spatial-multiplexing MIMO system that assists in recovering the linear combination of intended and unintended symbols from the transmitter and relay. The generalization of the proposed scheme using the Alamouti space-time relay transmission technique is briefly discussed in Section 5. Section 6 describes the K -user relay-aided MIMO interference channel. Section 7 presents the numerical results and the paper is concluded in Section 8.

## 2. Channel Model and Formula Definitions

**Figure 1:**
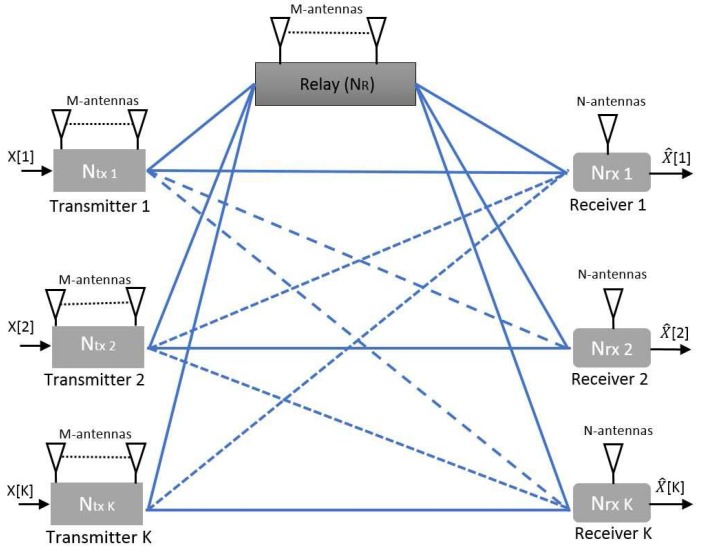
The K -user multiple-input-multiple-output (MIMO) ineterfrence channel model with a shared relay.

The Gaussian K -user MIMO downlink relay network channel model is depicted in Figure 1. K -user BS share information with the associated users through a shared relay.

We consider $N_{tx,k}$ transmitters and $N_{R}$ relay with *M* antennas transmits independent messages to the *N* antenna $N_{rx,k}$ receivers, where M≥k and k∈{1,2,3,…,K} . Consider that the communication over the relay functions in a half-duplex amplify and forward mode and sends an amplified version of the transmitted signal to the receiver with a processing delay. $X^{\left[k\right]}\left[n\right]\in C^{N_{\mathrm{tx},k}\times 1}$ and $X^{\left[R\right]}\left[n\right]\in C^{N_{R}\times 1}$ are the signal vectors from the transmitter and relay, respectively, where $h^{K}{\left[n\right]}^{T}=\left[h_{1}^{k}\left[n\right],\ldots ,h_{N_{tx,k}}^{k}\left[n\right]\right]$ and $h^{R}{\left[n\right]}^{T}=\left[h_{1}^{R}\left[n\right],\ldots ,h_{N_{R}}^{R}\left[n\right]\right]$ are the channel vectors from the transmitter and relay, respectively. We assume that the elements of the channel matrix, $H^{\left[R,K\right]}\left(n\right)$ , are drawn from an independent and identically distributed (i.i.d) complex Gaussian with zero mean and unit variance. The input–output relationships can be given as follows:

(1) $$\begin{array}{ccc} y_{j}^{k}\left(n\right)=\underset{\mathrm{Desired}\;\mathrm{signal}}{\underset{︸}{h_{j,j}^{{\left[k,k\right]}^{T}}\left(n\right)x_{j}^{k}\left(n\right)+h_{j,j}^{{\left[k,R\right]}^{T}}\left(n\right)x_{j}^{R}\left(n\right)}} & + & \underset{\mathrm{Interference}}{\underset{︸}{\sum_{j=1,j\neq k}^{K}h_{j,i}^{{\left[k,j\right]}^{T}}\left(n\right)x_{i}^{j}\left(n\right)}}+Z_{j}^{\left[k\right]}\left(n\right), \end{array}$$

(2) $$\begin{array}{ccc} Y_{j}^{R}\left(n\right) & = & \underset{\mathrm{Relay}\;\mathrm{signal}}{\underset{︸}{h_{j}^{\left[R,k\right]}\left(n\right)x_{j}^{k}\left(n\right)}}+Z_{j}^{\left[R\right]}\left(n\right), \end{array}$$

where $y_{j}^{k}\left(n\right)$ and $Y_{j}^{R}\left(n\right)$ are the *k*th receiver and relay signals, respectively, from the channel use n . $h_{j,j}^{{\left[k,k\right]}^{T}}\left(n\right)$ , $h_{j,j}^{{\left[k,R\right]}^{T}}\left(n\right)$ , and $h_{j,i}^{{\left[k,j\right]}^{T}}\left(n\right)$ represent the transmitter, relay, and interference channel matrices, respectively. $x_{j}^{k}\left(n\right)$ and $x_{j}^{R}\left(n\right)$ are the transmitter and relay signals; $Z_{j}^{\left[k\right]}\left(n\right)$ and $Z_{j}^{\left[R\right]}$ are the zero-mean additive white Gaussian noise signals for the *k*th receiver and relay, respectively.

### Key Definitions

**Definition** **1.** *Alamouti code (space time block code).*

*It is a technique used in wireless communication to transmit multiple data streams using massive antennas at the BS and a relay to exploit several received versions of data for improving the data transfer consistency and desired signal information [5,19].*

*Example: Alamouti code for a 2×2 MIMO wireless system model is given by*

(3) $$\begin{array}{c} \left[\begin{array}{c} y_{\left[jj\right]}\\ y_{\left[ji\right]}\\ y_{\left[ij\right]}^{*}\\ y_{\left[ii\right]}^{*} \end{array}\right]=\left[\begin{array}{c} h_{\left[jj\right]}\;h_{\left[ji\right]}\\ h_{\left[ij\right]}\;h_{\left[ii\right]}\\ h_{\left[ji\right]}^{*}\;-h_{\left[jj\right]}^{*}\\ h_{\left[ii\right]}^{*}\;-h_{\left[ij\right]}^{*} \end{array}\right]\left[\begin{array}{c} x_{j}^{k}\left[n\right]\\ x_{i}^{k}\left[n\right] \end{array}\right]+\left[\begin{array}{c} Z_{\left[jj\right]}\\ Z_{\left[ji\right]}\\ Z_{\left[ij\right]}^{*}\\ Z_{\left[ii\right]}^{*} \end{array}\right], \end{array}$$

*where ${\left[y_{jj},y_{ii}\right]}^{T}$ , and ${\left[y_{ij},y_{ji}\right]}^{T}$ denote the received, desired, and interference signal vectors. $h_{jj}$ , $h_{ii}$ , and $h_{ji}$ , $h_{ij}$ are the desired and interference channel matrices, where $x_{j}^{k}\left[n\right]$ , $x_{i}^{k}\left[n\right]$ denote the transmitted signals; ${\left[Z_{jj},Z_{ji}\right]}^{T}$ , ${\left[Z_{ij},Z_{ii}\right]}^{T}$ denote the zero-mean additive white Gaussian Noise (AWGN).*

**Definition** **2.** *(Degree of freedom and achievable sum-rate).*

*The degree of freedom (DoF) region for this network topology is $d_{1}+d_{2}+,\ldots ,+d_{n}\leq 1$ and, for a symmetric network, it is $d_{sym}=\frac{1}{n}$ [3,20]*

(4) $$\begin{array}{c} d_{sym}=\underset{P\to \infty }{\lim\;\sup}\underset{(R,\ldots ,R)\in C}{\sup}\frac{R\sum (P)}{\log P},\\ D\;=\;\{\left(d_{1},\ldots ,d_{k}\right)\;\in \;R_{+}\;:\;d_{i}\;=\;\underset{P\to \infty }{\lim\;\sup}\frac{R_{i}\sum \left(P\right)}{\log P},\forall_{i}\\ s.t(R_{1},\ldots ,R_{k})\in C\}. \end{array}$$

*The achievable rate for the transmitter and receiver pairs defined for the K th user is*

(5) $$\begin{array}{c} r^{\left[k\right]}=\underset{P\to \infty }{\lim\;\sup}\frac{R^{\left[k\right]}\left(P\right)}{\log(P)}. \end{array}$$

*The achievable sum-rate for the proposed K -user MIMO relay interference channel model [16,21] is expressed as*

(6) $$\begin{array}{c} R_{K}\;\leq \log\left(1+\frac{∥h_{j,j}^{{\left[k,k\right]}^{T}}\left(n\right){∥}^{2}P+∥h_{R_{j}}^{{\left[k,R\right]}^{T}}\left(n\right){∥}^{2}\left(\frac{P_{R}}{2}\right)}{∥h_{j,i}^{{\left[k,j\right]}^{T}}\left(n\right){∥}^{2}P+1}+\frac{2\alpha ∥h_{j,j}^{{\left[k,k\right]}^{T}}\left(n\right)∥∥h_{R_{j}}^{{\left[k,R\right]}^{T}}\left(n\right)∥\sqrt{P(\frac{P_{R}}{2})}}{∥h_{j,i}^{{\left[k,j\right]}^{T}}\left(n\right){∥}^{2}P+1}\right). \end{array}$$

All the users (nodes) are subject to an average power-constraint (P) is defined as $P=SNR\times \sigma^{2}$ and $R^{\left[k\right]}\left(P\right)$ indicate the achievable rate, where (SNR) represents signal-to-noise ratio [22]. The transmitted signals, $E(∥X^{\left[k\right]}\left[n\right]{∥}^{2})\leq P$ and the relay signals, $E(∥X^{\left[R\right]}\left[n\right]{∥}^{2})\leq P_{R}$ , subject to the average power constraint are briefly described, where the coefficient α represents the in-phase addition of signals coming from the transmitters and the relay. The fraction of the interference power for the desired signal is defined as follows:

(7) $$\begin{array}{ccc} q_{avg} & = & \frac{1}{k}\sum_{k=1}^{k}\sigma_{k}. \end{array}$$

## 3. Three-User MIMO Relay-Aided Broadcast Channel

**Figure 2:**
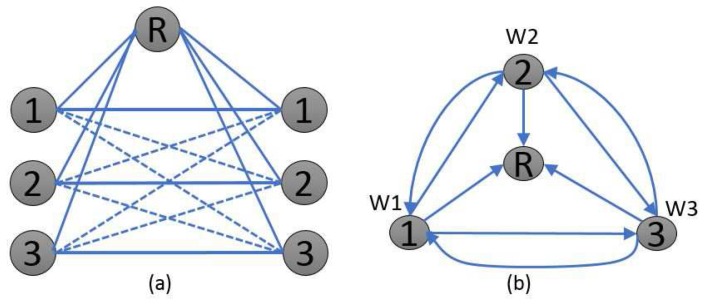
(**a**) simple three-user network topology with a relay and (**b**) side information conflict diagram.

**Figure 3:**
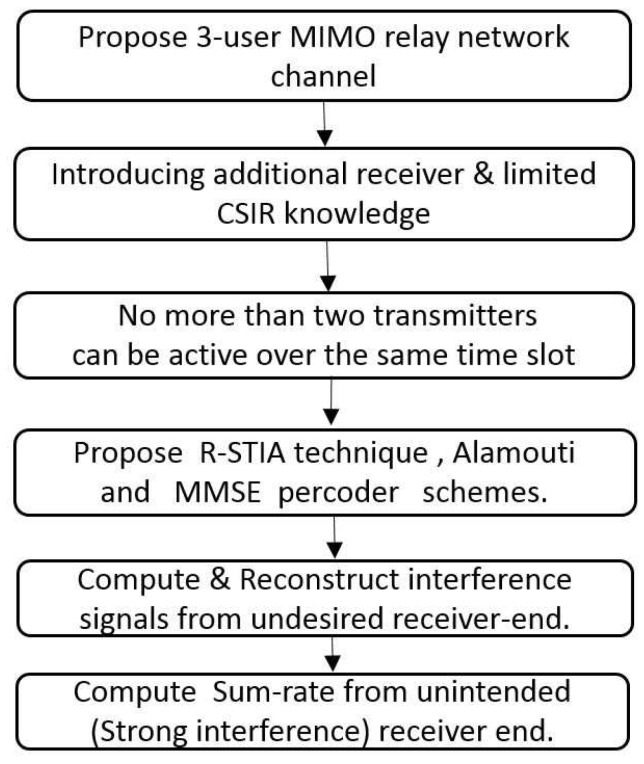
Proposed optimization framework.

In this section, we propose a simple three-user network topology with a shared relay, as shown in Figure 2. We summarize the important steps in the proposed optimization framework scheme shown in Figure 3. We have considered K = 3 users, where the transmitter, $N_{tx,k}$ and the relay, $N_{R}$ , have massive antennas and the receiver, $N_{rx,k}$ , has a single antenna [23]. We primarily focus on scenarios in which the CSIT is not known at the receiver-side, where it has access to limited CSIR knowledge only:

(8) $$\begin{array}{c} {\hat{X}}^{\left[k\right]}\left[n\right]=\sum_{k=1}^{3}h_{n}^{\left[k\right]}\left[k\right]u_{n}^{\left[k\right]}. \end{array}$$

Based on this space-time relay beamforming method, the relay forwards the received signals, while all of the three transmitters remain silent. More specifically, the space-time relay beamforming matrix can carry only two symbols for the receiver, K∈{1,2,3} , which can be denoted by $V^{\left[k\right]}=\left[V_{k}^{\left[1\right]},V_{k}^{\left[2\right]},V_{k}^{\left[3\right]}\right]\in C^{3\times 3}$ and the transmitter signal vector at the relay is given by

(9) $$\begin{array}{cc} \hat{X}{\;}^{\left[R\right]}\left[n\right] & \;=\;\beta \left(V_{1}^{\left[1\right]}{\hat{u}}_{1}^{\left[1\right]}+V_{2}^{\left[1\right]}{\hat{u}}_{2}^{\left[1\right]}\right)\;\;\;+\;\beta \left(V_{1}^{\left[2\right]}{\hat{u}}_{1}^{\left[2\right]}\;\;\;+\;\;\;V_{2}^{\left[2\right]}{\hat{u}}_{2}^{\left[2\right]}\right)\;\;\;+\;\;\beta \left(V_{1}^{\left[3\right]}{\hat{u}}_{1}^{\left[3\right]}\;\;+\;\;V_{2}^{\left[3\right]}{\hat{u}}_{2}^{\left[3\right]}\;\right),\\ & \;=\;\beta V_{1}^{\left[1\right]}u_{1}^{\left[1\right]}+\beta V_{2}^{\left[1\right]}u_{2}^{\left[1\right]}\;+\;\;\;\beta V_{1}^{\left[2\right]}u_{1}^{\left[2\right]}\;\;\;+\;\;\beta V_{2}^{\left[2\right]}u_{2}^{\left[2\right]}\;\;\;+\;\beta V_{1}^{\left[3\right]}u_{1}^{\left[3\right]}\;\;\;+\;\;\beta V_{2}^{\left[3\right]}u_{2}^{\left[3\right]}\;\;, \end{array}$$

where β is the power normalization factor. ${\tilde{x}}^{\left[k\right]}\left[n\right]$ and ${\tilde{x}}^{\left[R\right]}\left[n\right]$ are the original symbol vectors for the transmitter and relay, respectively. Although the pre-equalization can be characterized by a pre-equalizer weight matrix, $W\in C^{N_{tx,k}\times N_{tx,k}}$ and the precoding symbol vector, *x*, in terms of W , can be defined as $x=W\hat{x}$ . We provide a refined analysis of the corresponding weight matrix, described under the MMSE pre-equalization.

The total Gaussian point-to-point interfering with the multiple access relay channel (PIMAC) power constraints after pre-equalization can be expressed as follows

(10) $$\begin{array}{c} E\left[tr\left(x^{\left[R\right]}\left(n\right)x^{{\left[R\right]}^{†}}\left(n\right)\right)\right]\leq P, \end{array}$$

where † represents the conjugate transpose and the power normalization factor β is expressed as

(11) $$\begin{array}{c} \beta =\;\sqrt{\frac{P}{E\{Tr\left(H\hat{u}{\hat{u}}^{†}H^{-1}\right)\}}}. \end{array}$$

Furthermore, the MMSE pre-equalization weight matrix is selected as

(12) $$\begin{array}{cc} W_{\mathrm{MMSE}} & \;=\;\beta \;\;\;\times \;\;argmin\;E\left[∥\beta^{-1}\left(HW\hat{x}\right)-\hat{x}{∥}^{2}\right],\\ & \;=\;\beta \times H^{H}{\left(HH^{H}+\frac{\sigma_{z}^{2}}{\sigma_{x}^{2}}I\right)}^{-1}. \end{array}$$

## 4. Precoded Spatial-Multiplexing MIMO Systems

In this section, we focus on a precoded spatial-multiplexing MIMO system to transmit the independent and separately encoded desired signals using multiple antennas at the transmitters and relay, where the common relay shares limited CSIR knowledge with the receivers, as shown in Figure 2. The three key components in relay aided three-user MIMO broadcast relay channels are as follows.

**Figure 4:**
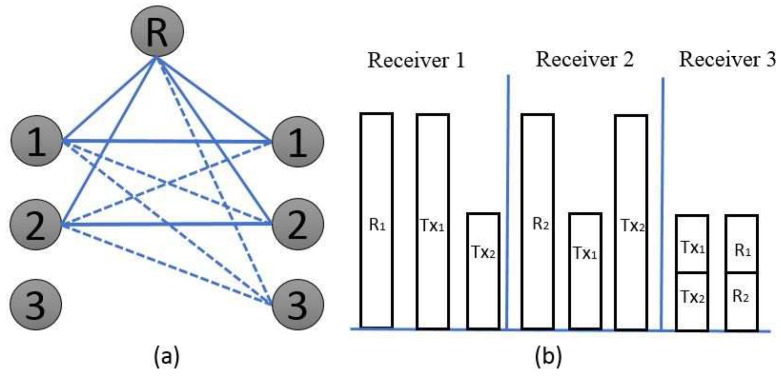
(**a**) simple network topology over the first time slot and (**b**) block representation of the received signal.

**Figure 5:**
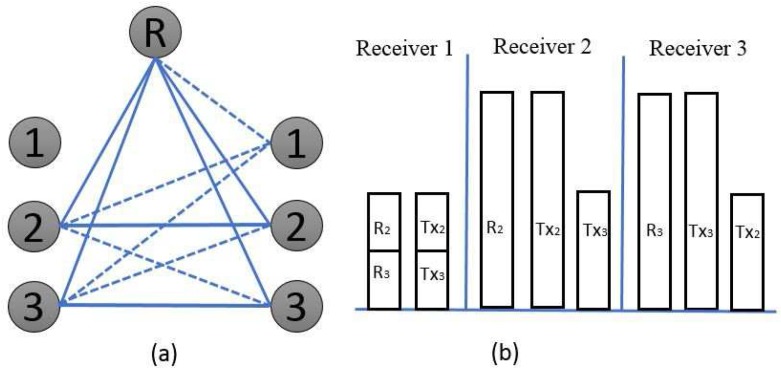
(**a**) simple network topology over the second time slot and (**b**) block representation of the received signal.

**Figure 6:**
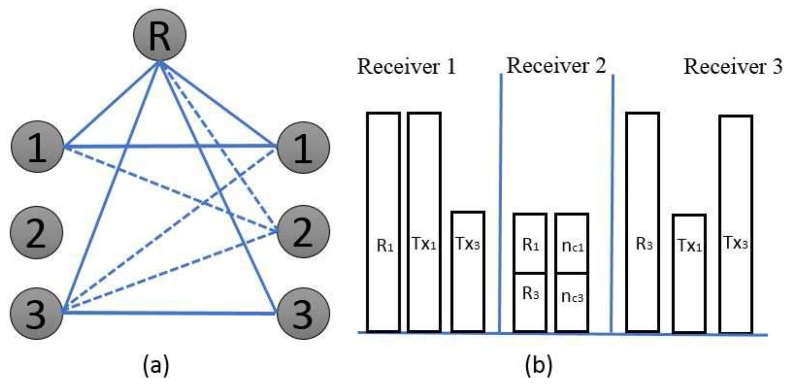
(**a**) simple network topology over the third time slot and (**b**) block representation of the received signal.

The first key component initializes the three time slots in which each user sends two independent intended symbols, $u_{1}^{\left(K\right)}$ and $u_{2}^{\left(K\right)}$ , using two antennas. Over each of the three time slots, the corresponding network topology of the active transmitters and the block representation of the received signals are illustrated in Figure 4, Figure 5 and Figure 6, respectively. In time slot-1, the user-1 transmitter signal is $X^{\left[1\right]}\left[1\right]={\left[u_{1}^{\left(1\right)},u_{2}^{\left(1\right)}\right]}^{T}$ , where $u_{1}^{\left(1\right)}$ and $u_{2}^{\left(1\right)}$ are the respective intended symbols. In time slot-2, the user-2 transmitter signal is $X^{\left[2\right]}\left[2\right]={\left[u_{1}^{\left(2\right)},u_{2}^{\left(2\right)}\right]}^{T}$ , where $u_{1}^{\left(2\right)}$ and $u_{2}^{\left(2\right)}$ are the respective intended symbols. In time slot-3, the user-3 transmitter signal is $X^{\left[3\right]}\left[3\right]={\left[u_{1}^{\left(3\right)},u_{2}^{\left(3\right)}\right]}^{T}$ , where $u_{1}^{\left(3\right)}$ and $u_{2}^{\left(3\right)}$ are the corresponding intended symbols. The transmitter corresponding to each of the above-mentioned time slots, {K=1,2,3}, sends two independent symbols for sharing the information with the intended receiver [1]. Thus, three transmitters belonging to three time slots each send a total of six independent symbols. The intended symbols are indicated by $F^{\left[k,i\right]}\left[W_{n}\right]$ , which express the linear combination of the transmitting signals from user K .

**Algorithm 1** Multiuser MIMO Scheduling.
Step:1
.  
Initialize
:

K∈{1,2,…,k}
; 
$V^{\left[k\right]}\;=\;\left[V_{k}^{\left[1\right]},V_{k}^{\left[2\right]},\ldots ,V_{k}^{\left[k\right]}\right]$
;
Transmitter & relay signal: 
${\hat{X}}^{\left[k\right]}\left[n\right]\;\;\;\;=\;\;\;\sum_{k=1}^{k}h_{c_{n}}^{\left[k\right]}\left[k\right]u_{n}^{\left[k\right]};$


$\hat{X}{\;}^{\left[R\right]}\left[n\right]\;\;\;=\;\;\;\beta \left(V_{k}^{\left[1\right]}{\hat{u}}_{1}^{\left[k\right]}+V_{k}^{\left[1\right]}{\hat{u}}_{2}^{\left[k\right]}\right)$
; 
$\beta \;\;\;=\;\;\;\sqrt{\frac{P}{E\{Tr\left(H\hat{u}{\hat{u}}^{†}H^{-1}\right)\}}}$
.

Step:2
.  
Repeat
:
1:**for** k=1,2,3 **do**2:Compute the transmitter & relay signals at the unintended receiver:

$$F^{\left[3,3\right]}\left[W_{3}\right]=h_{\left[c_{3}\right]}^{\left[3\right]}\left[k\right]u_{1}^{\left[k\right]}+h_{\left[c_{3}\right]}^{\left[k\right]}\left[k\right]u_{2}^{\left[k\right]},$$


$$F^{\left[2,2\right]}\left[W_{2}\right]=\;\;\;h_{\left[c_{2}\right]}^{\left[2\right]}\left[k\right]u_{1}^{\left[k\right]}+\;\;\;h_{\left[c_{2}\right]}^{\left[k\right]}\left[k\right]u_{2}^{\left[k\right]},$$


$$\left[\begin{array}{c} \beta h_{\left[R_{3}\right]}^{(3,R)T}\left[1\right]\\ \beta h_{\left[R_{2}\right]}^{(2,R)T}\left[1\right] \end{array}\right]V^{\left(k\right)}\left[n\right],$$
3:User-1 reconstructed signal from the transmitter and relay. Decoding: $y_{\left(1\right)}^{\left[1\right]}\;=\;$ $y_{\left(3\right)}^{\left[1\right]}+y_{\left(2\right)}^{\left[1\right]}⟹$


$$=\;\;\;\left(h_{\left[R_{1}\right]}^{{\left[1,R\right]}^{T}}\;\;\;\;\left(3\right)+h_{\left[R_{2}\right]}^{{\left[1,R\right]}^{T}}\;\;\;\;\left(3\right)\right)\;X^{\left[R\right]}\;\left[3\right]+\left(h_{\left[R_{3}\right]}^{{\left[1,R\right]}^{T}}\;\;\;\;\left(2\right)+h_{\left[R_{1}\right]}^{{\left[1,R\right]}^{T}}\;\;\;\;\left(2\right)\right)\;X^{\left[R\right]}\;\left[2\right].$$


$$=-\left\{h_{\left[c_{2}\right]}^{{\left[1,1\right]}^{*}}\;\;\left(2\right)+h_{\left[c_{3}\right]}^{\left[1,1\right]}\;\;\left(3\right)\right\}u_{1}^{\left[1\right]}+\left\{h_{\left[c_{1}\right]}^{{\left[1,1\right]}^{*}}\;\;\left(1\right)+h_{\left[c_{1}\right]}^{\left[1,1\right]}\;\;\left(1\right)\right\}u_{2}^{\left[1\right]}.$$
4:Achievable Sum-rate for K-user MIMO relay channel:

$$R_{K}\leq \log\left(1+\frac{∥h_{j,j}{∥}^{2}P+∥h_{R_{j}}{∥}^{2}\left(\frac{P_{R}}{2}\right)}{∥h_{j,i}{∥}^{2}P+1}+\frac{2\alpha ∥h_{j,j}∥∥h_{R_{j}}∥\sqrt{P(\frac{P_{R}}{2})}}{∥h_{j,i}{∥}^{2}P+1}\right)$$
5:**end for**.
for fixed 
${\hat{X}}^{\left[k],[R\right]}$
 compute 
$y_{\left(2\right)}^{\left[2\right]},y_{\left(3\right)}^{\left[3\right]}$
 until recovered.


The second key component is the multiuser MIMO scheduling Algorithm 1, which forces the active transmitters to be assigned during the time slots and provides the basic initialization necessary to recover the interference signals from the unintended receiver. In three time slots, the linear combination of the desired signal information is reconstructed from the unintended receiver.

The third main component initializes the channel coefficients to identify the corresponding interference signals from the relay and the transmitters. These linear combinations of the desired signal information enable the reconstruction of the interference signals from the unintended receiver in which $\;Z^{\left[k\right]}\left(t\right)$ is a realization of random variables for user K={1,2,3} . $Z^{\left[k\right]}∼CN\left(0,1\right)$ impersonates the additive white Gaussian noise signal; the corresponding complex (static) channel coefficients [24,25] are $h_{k}\;\;=\;\;\left\{R_{1},R_{2},R_{3},d_{1},d_{2},d_{3},c_{1},c_{2},c_{3}\right\}$ , where $R_{k},d_{k},c_{k}$ represent the relay signals, the directly transmitted signals, and the interference signals, respectively. In the Gaussian linear deterministic PIMAC models depicted in Figure 4, Figure 5 and Figure 6, the corresponding transmitter binary vectors are $x_{i}\;=\;F_{2}^{q}$ , where $q=max\{n_{R_{1}},n_{R_{2}},n_{R_{3}},n_{d_{1}},n_{d_{2}},n_{d_{3}},n_{c_{1}},n_{c_{2}},n_{c_{3}}\}$ and the integer of $n_{k}$ represents the additive white Gaussian channel coefficients, $n_{k}=\lfloor \log_{2}\left(P\right|h_{k}{|}^{2}\rfloor$ . We assume that all of the nodes have global CSIR knowledge, where the noise, $Z^{\left[k\right]}\left[t\right]$ , is independent and identically distributed (i.i.d) over these time slots.

**Over the first time slot**: Transmitters 1 and 2 are kept active because transmitter 3 is made silent, as shown in Figure 4. Under such conditions, receiver 1, $F^{\left[1,1\right]}\left[W_{1}\right]$ and receiver 2, $F^{\left[2,2\right]}\left[W_{1}\right]$ , can acquire the superposition of the two desired symbols. Nevertheless, receiver 3 can eavesdrop on the linear combination of the unintended interference signal from the active transmitters:

(13) $$\begin{array}{c} F^{\left[1,1\right]}\left[W_{1}\right]=h_{\left[c_{1}\right]}^{\left[1,1\right]}\left[1\right]u_{1}^{\left[1\right]}+h_{\left[c_{1}\right]}^{\left[1,1\right]}\left[1\right]u_{2}^{\left[1\right]},\\ F^{\left[2,2\right]}\left[W_{1}\right]=h_{\left[c_{2}\right]}^{\left[2,2\right]}\left[1\right]u_{1}^{\left[1\right]}+h_{\left[c_{2}\right]}^{\left[2,2\right]}\left[1\right]u_{2}^{\left[1\right]}. \end{array}$$

In order to deliver the same linear combination of undesired signals from receiver 3, the principal idea of the proposed beamforming scheme is to select the beamforming matrix from transmitters 1 and 2 with a shared relay:

(14) $$\begin{array}{c} \left[\begin{array}{c} \beta h_{\left[R_{1}\right]}^{(1,R)T}\left[n\right]\\ \beta h_{\left[R_{2}\right]}^{(2,R)T}\left[n\right] \end{array}\right]V^{\left(3\right)}\left[n\right]=\left[\begin{array}{c} h_{\left[c_{1}\right]}^{(1,1)T}\left[n\right]\\ h_{\left[c_{2}\right]}^{(2,2)T}\left[n\right] \end{array}\right]. \end{array}$$

**Over the second time slot**: Transmitters 2 and 3 are kept active, whereas, transmitter 1 is made silent, as shown in Figure 5. The concept is identical as discussed for time slot 1; receiver 2, $F^{\left[2,2\right]}\left[W_{2}\right]$ and receiver 3 , $F^{\left[3,3\right]}\left[W_{2}\right]$ , can attain the superposition of the two desired symbols because receiver 1 can eavesdrop on the linear combination of the unintended interference signal from the active transmitters:

(15) $$\begin{array}{c} F^{\left[2,2\right]}\left[W_{2}\right]=h_{\left[c_{2}\right]}^{\left[2,2\right]}\left[2\right]u_{1}^{\left[2\right]}+h_{\left[c_{2}\right]}^{\left[2,2\right]}\left[2\right]u_{2}^{\left[2\right]},\\ F^{\left[3,3\right]}\left[W_{2}\right]=h_{\left[c_{3}\right]}^{\left[3,3\right]}\left[2\right]u_{1}^{\left[2\right]}+h_{\left[c_{3}\right]}^{\left[3,3\right]}\left[2\right]u_{2}^{\left[2\right]}. \end{array}$$

To deliver the same linear combination of undesired signals from receiver 1, the beamforming matrix is selected from transmitters 2 and 3 with a shared relay:

(16) $$\begin{array}{c} \left[\begin{array}{c} \beta h_{\left[R_{2}\right]}^{(2,R)T}\left[n\right]\\ \beta h_{\left[R_{3}\right]}^{(3,R)T}\left[n\right] \end{array}\right]V^{\left(1\right)}\left[n\right]=\left[\begin{array}{c} h_{\left[c_{2}\right]}^{(2,2)T}\left[n\right]\\ h_{\left[c_{3}\right]}^{(3,3)T}\left[n\right] \end{array}\right]. \end{array}$$

**Over the third time slot**: In addition, for this time slot, we keep transmitters 3 and 1 active because transmitter 2 is kept in a silent mode, as shown in Figure 6. Receiver 3, $F^{\left[3,3\right]}\left[W_{3}\right]$ and receiver 1, $F^{\left[1,1\right]}\left[W_{3}\right]$ , can attain the superposition of the two desired symbols because receiver 2 can eavesdrop on the linear combination of the unintended interference signal from the active transmitters:

(17) $$\begin{array}{c} F^{\left[3,3\right]}\left[W_{3}\right]=h_{\left[c_{3}\right]}^{\left[3,3\right]}\left[3\right]u_{1}^{\left[3\right]}+h_{\left[c_{3}\right]}^{\left[3,3\right]}\left[3\right]u_{2}^{\left[3\right]},\\ F^{\left[1,1\right]}\left[W_{3}\right]=h_{\left[c_{1}\right]}^{\left[1,1\right]}\left[3\right]u_{1}^{\left[3\right]}+h_{\left[c_{1}\right]}^{\left[1,1\right]}\left[3\right]u_{2}^{\left[3\right]}. \end{array}$$

To deliver the same linear combination of undesired signals from receiver 2, the beamforming matrix is selected from transmitters 3 and 1 with a shared relay:

(18) $$\begin{array}{c} \left[\begin{array}{c} \beta h_{\left[R_{3}\right]}^{(3,R)T}\left[n\right]\\ \beta h_{\left[R_{1}\right]}^{(1,R)T}\left[n\right] \end{array}\right]V^{\left(2\right)}\left[n\right]=\left[\begin{array}{c} h_{\left[c_{3}\right]}^{(3,3)T}\left[n\right]\\ h_{\left[c_{1}\right]}^{(1,1)T}\left[n\right] \end{array}\right]. \end{array}$$

During all three of the time slots, the relay approximates the received transmitter symbols by multiplying the receiver beamforming matrix using the two received symbols [2]:

(19) $$\begin{array}{cc} {\hat{S}}^{\left[k\right]} & =\left[\begin{array}{c} {\hat{u}}_{1}^{\left[k\right]}\\ {\hat{u}}_{2}^{\left[k\right]} \end{array}\right]\;\;\;={\left(H_{\left[c_{k}\right]}^{\left[R,k\right]}\left(k\right)\right)}^{-1}\;\;y^{\left[R\right]}\left(k\right),\\ & =u^{\left[k\right]}\;\;\;+\;\;\;{\left(H_{\left[c_{k}\right]}^{\left[R,k\right]}\left(k\right)\right)}^{-1}\;\;\;\;,\;k\in 1,2,3. \end{array}$$

## 5. Space-Time Relay Transmission

Space-time relay transmission efficiently forwards the desired signals through multiple transmitters and a shared relay to the destination. In all three of the time slots considered in Figure 4, Figure 5 and Figure 6, the receiver must use the Alamouti code to decode the interference signals. We know that the relay has a two-phase protocol and, during the first phase of the protocol, the relay receives the source broadcasted signals from multiple transmitters, while, in the second phase of the protocol, the relay transmits source signals to the destinations.

By utilizing the Alamouti method for equations (13) and (14) in time slot 1, the interference signal from the relay and transmitters can be communicated as

(20) $$\begin{array}{c} V^{\left[3\right]}\;\;=\;\;\;\frac{1}{\beta }\;\;\underset{\mathrm{relay}\;\mathrm{channel}\;\mathrm{vector}}{\underset{︸}{{\left[\begin{array}{c} h_{\left[R_{1}\right]}^{\left[1,R\right]}\left[3\right]\;h_{\left[R_{2}\right]}^{\left[1,R\right]}\left[3\right]\\ h_{\left[R_{1}\right]}^{\left[2,R\right]}\left[3\right]\;h_{\left[R_{2}\right]}^{\left[2,R\right]}\left[3\right] \end{array}\right]}^{-1}\;\;\;}}\underset{\mathrm{transmitter}\;\mathrm{channel}\;\mathrm{vector}}{\underset{︸}{\left[\begin{array}{c} -h_{\left[c_{2}\right]}^{{\left[1,1\right]}^{*}}\left[2\right]\;h_{\left[c_{1}\right]}^{{\left[1,1\right]}^{*}}\left[1\right]\\ h_{\left[c_{2}\right]}^{\left[2,2\right]}\left[1\right]\;h_{\left[c_{1}\right]}^{\left[2,2\right]}\left[2\right] \end{array}\right]\;\;}}\;\;\;. \end{array}$$

The corresponding transmitter 1 and 2 signals, recovered during time slot 1 from the unintended receiver 3 can be expressed as

(21) $$\begin{array}{cc} y_{\left(3\right)}^{\left[1\right]} & =\;\left(h_{\left[R_{1}\right]}^{{\left[1,R\right]}^{T}}\left(3\right)+h_{\left[R_{2}\right]}^{{\left[1,R\right]}^{T}}\left(3\right)\right)X^{\left[R\right]}\left[3\right]+Z^{\left[1\right]}\left(3\right),\\ & =-h_{\left[c_{2}\right]}^{{\left[1,1\right]}^{*}}\left(2\right)u_{1}^{\left[1\right]}+h_{\left[c_{1}\right]}^{{\left[1,1\right]}^{*}}\left(1\right)u_{2}^{\left[1\right]}+Z^{\left[1\right]}\left(3\right). \end{array}$$

(22) $$\begin{array}{cc} y_{\left(3\right)}^{\left[2\right]} & =\;\left(h_{\left[R_{1}\right]}^{{\left[2,R\right]}^{T}}\left(3\right)+h_{\left[R_{2}\right]}^{{\left[2,R\right]}^{T}}\left(3\right)\right)X^{\left[R\right]}\left[3\right]+Z^{\left[2\right]}\left(3\right),\\ & =\;h_{\left[c_{2}\right]}^{\left[2,2\right]}\left(2\right)u_{1}^{\left[2\right]}+h_{\left[c_{1}\right]}^{\left[2,2\right]}\left(1\right)u_{2}^{\left[2\right]}+Z^{\left[2\right]}\left(3\right). \end{array}$$

Equivalently, Equations (15) and (16) are expressed for time slot 3, as mentioned below:

(23) $$\begin{array}{c} V^{\left[1\right]}\;\;=\;\;\;\frac{1}{\beta }\;\;\underset{\mathrm{relay}\;\mathrm{channel}\;\mathrm{vector}}{\underset{︸}{{\left[\begin{array}{c} h_{\left[R_{2}\right]}^{\left[2,R\right]}\left[1\right]\;h_{\left[R_{3}\right]}^{\left[2,R\right]}\left[1\right]\\ h_{\left[R_{2}\right]}^{\left[3,R\right]}\left[1\right]\;h_{\left[R_{3}\right]}^{\left[3,R\right]}\left[1\right] \end{array}\right]}^{-1}\;\;\;}}\underset{\mathrm{transmitter}\;\mathrm{channel}\;\mathrm{vector}}{\underset{︸}{\left[\begin{array}{c} -h_{\left[c_{3}\right]}^{{\left[2,2\right]}^{*}}\left[3\right]\;h_{\left[c_{2}\right]}^{{\left[2,2\right]}^{*}}\left[2\right]\\ \;h_{\left[c_{2}\right]}^{\left[3,3\right]}\left[2\right]\;h_{\left[c_{3}\right]}^{\left[3,3\right]}\left[3\right] \end{array}\right]\;\;}}\;\;\;. \end{array}$$

The corresponding transmitter 2 and 3 signals, recovered during time slot 2 from the unintended receiver 1, can be expressed as

(24) $$\begin{array}{cc} y_{\left(1\right)}^{\left[2\right]} & =\;\left(h_{\left[R_{2}\right]}^{{\left[2,R\right]}^{T}}\left(1\right)+h_{\left[R_{3}\right]}^{{\left[2,R\right]}^{T}}\left(1\right)\right)X^{\left[R\right]}\left[1\right]+Z^{\left[2\right]}\left(1\right),\\ & =-h_{\left[c_{3}\right]}^{{\left[2,2\right]}^{*}}\left(3\right)u_{1}^{\left[2\right]}+h_{\left[c_{2}\right]}^{{\left[2,2\right]}^{*}}\left(2\right)u_{2}^{\left[2\right]}+Z^{\left[2\right]}\left(1\right). \end{array}$$

(25) $$\begin{array}{cc} y_{\left(1\right)}^{\left[3\right]} & =\;\left(h_{\left[R_{2}\right]}^{{\left[3,R\right]}^{T}}\left(1\right)+h_{\left[R_{3}\right]}^{{\left[3,R\right]}^{T}}\left(1\right)\right)X^{\left[R\right]}\left[1\right]+Z^{\left[3\right]}\left(1\right),\\ & =\;h_{\left[c_{2}\right]}^{\left[3,3\right]}\left(2\right)u_{1}^{\left[3\right]}+h_{\left[c_{3}\right]}^{\left[3,3\right]}\left(3\right)u_{3}^{\left[2\right]}+Z^{\left[3\right]}\left(1\right). \end{array}$$

Similarly, Equations (17) and (18) are represented for time slot 3, as mentioned below:

(26) $$\begin{array}{c} V^{\left[2\right]}\;\;=\;\;\;\frac{1}{\beta }\;\;\underset{\mathrm{relay}\;\mathrm{channel}\;\mathrm{vector}}{\underset{︸}{{\left[\begin{array}{c} h_{\left[R_{3}\right]}^{\left[3,R\right]}\left[2\right]\;h_{\left[R_{1}\right]}^{\left[3,R\right]}\left[2\right]\\ h_{\left[R_{3}\right]}^{\left[1,R\right]}\left[2\right]\;h_{\left[R_{1}\right]}^{\left[1,R\right]}\left[2\right] \end{array}\right]}^{-1}\;\;\;}}\underset{\mathrm{transmitter}\;\mathrm{channel}\;\mathrm{vector}}{\underset{︸}{\left[\begin{array}{c} -h_{\left[c_{1}\right]}^{{\left[3,3\right]}^{*}}\;\;\left[1\right]\;h_{\left[c_{3}\right]}^{{\left[3,3\right]}^{*}}\;\left[3\right]\\ h_{\left[c_{3}\right]}^{\left[1,1\right]}\left[3\right]\;h_{\left[c_{1}\right]}^{\left[1,1\right]}\left[1\right] \end{array}\right]\;\;}}\;\;\;. \end{array}$$

The corresponding transmitter 3 and 1 signals, recovered during time slot 2 from the unintended receiver 2, can be expressed as

(27) $$\begin{array}{cc} y_{\left(2\right)}^{\left[3\right]} & =\;\left(h_{\left[R_{3}\right]}^{{\left[3,R\right]}^{T}}\left(2\right)+h_{\left[R_{1}\right]}^{{\left[3,R\right]}^{T}}\left(2\right)\right)X^{\left[R\right]}\left[2\right]+Z^{\left[3\right]}\left(2\right),\\ & =\;-h_{\left[c_{1}\right]}^{{\left[3,3\right]}^{*}}\left(1\right)u_{1}^{\left[3\right]}+h_{\left[c_{3}\right]}^{{\left[3,3\right]}^{*}}\left(3\right)u_{2}^{\left[3\right]}+Z^{\left[3\right]}\left(2\right). \end{array}$$

(28) $$\begin{array}{cc} y_{\left(2\right)}^{\left[1\right]} & =\;\left(h_{\left[R_{3}\right]}^{{\left[1,R\right]}^{T}}\left(2\right)+h_{\left[R_{1}\right]}^{{\left[1,R\right]}^{T}}\left(2\right)\right)X^{\left[R\right]}\left[2\right]+Z^{\left[1\right]}\left(2\right),\\ & =\;h_{\left[c_{3}\right]}^{\left[1,1\right]}\left(3\right)u_{1}^{\left[1\right]}+h_{\left[c_{1}\right]}^{\left[1,1\right]}\left(1\right)u_{2}^{\left[1\right]}+Z^{\left[1\right]}\left(2\right). \end{array}$$

**Decoding**: The overall decoding process requires the separation of the user interference signals based on the CSIR knowledge at the receiver side. Consequently, the user 1 signal is decoded based on the relay and receiver signals from $y_{\left(1\right)}^{\left[1\right]}=y_{\left(3\right)}^{\left[1\right]}+y_{\left(2\right)}^{\left[1\right]}$ :

(29)y(3)[1]+y(2)[1]=(h[R1][1,R]T(3)+h[R2][1,R]T(3))X[R][3]+(h[R3][1,R]T(2)+h[R1][1,R]T(2))X[R][2]︸recoveredrelaysignal(30)=−h[c2][1,1]*(2)u1[1]+h[c1][1,1]*(1)u2[1]+h[c3][1,1](3)u1[1]+h[c1][1,1](1)u2[1]︸recoveredtransmittersignal.

Similarly, we can compute the received signals $y_{\left(2\right)}^{\left[2\right]}=y_{\left(3\right)}^{\left[2\right]}+y_{\left(1\right)}^{\left[2\right]}$ and $y_{\left(3\right)}^{\left[3\right]}=y_{\left(1\right)}^{\left[3\right]}+y_{\left(2\right)}^{\left[3\right]}$ .

After removing the corresponding interference signals, the effective channel input–output relationship for user 1 is as follows:

(31) $$\begin{array}{c} \underset{y_{1}^{\left[1\right]}}{\underset{︸}{\left[\begin{array}{c} y_{1}^{\left[1\right]}-y_{2}^{\left[2\right]}\\ y_{1}^{\left[1\right]}-y_{3}^{\left[3\right]} \end{array}\right]}}=\underset{y_{1}^{\left[1\right]}-y_{2}^{\left[2\right]},y_{1}^{\left[1\right]}-y_{3}^{\left[3\right]}}{\underset{︸}{\left[\begin{array}{c} \left(y_{\left(3\right)}^{\left[1\right]}+y_{\left(2\right)}^{\left[1\right]}\right)-\left(y_{\left(3\right)}^{\left[2\right]}+y_{\left(1\right)}^{\left[2\right]}\right)\\ \left(y_{\left(3\right)}^{\left[1\right]}+y_{\left(2\right)}^{\left[1\right]}\right)-\left(y_{\left(1\right)}^{\left[3\right]}+y_{\left(2\right)}^{\left[3\right]}\right) \end{array}\right]}}\;\;\;\underset{X_{1}^{\left[1\right]}}{\underset{︸}{\left[\begin{array}{c} u_{1}^{\left(1\right)}\\ u_{2}^{\left(1\right)} \end{array}\right]}}+\underset{z_{1}^{\left(1\right)}}{\underset{︸}{\left[\begin{array}{c} z_{1}^{\left(1\right)}-z_{2}^{\left(2\right)}\\ z_{1}^{\left(1\right)}-z_{3}^{\left(3\right)} \end{array}\right]}}. \end{array}$$

### Discussion

The proposed idea is to create a relay-aided network channel where each intended receiver experiences less interference and the unintended (additional) receiver experiences more interferences. Remarkably, the intended receivers have very weak interference, since intended receivers are affected by only one interference signal. The single unintended receiver has very strong interference because the unintended receiver is affected by two transmitter interference signals and one relay signal over the time slots. Moreover, this paper offers an efficient algorithm and a channel co-efficient for the desired, undesired, and relay signals for identifying and aligning the corresponding signals resourcefully. Subsequently, by introducing an additional (unintended) receiver at a very-strong interference region and with certain limited CSIR knowledge, the network settings are valid for recovering the combination of the three co-interference signals from transmitters and relay. By such R-STIA and Alamouti techniques, the interference signals are reconstructed at the unintended PIMAC receiver-end.

The authors in [26] proposed a Quasi Orthogonal (QO)-STIA technique for a two-user *X* channel, where the interference cancellation and alignment is based on only two intended receivers. The decoding complexity is reasonably high, since the receiver has to decode the intended symbols over each time slots until the desired signal is recovered. The authors in [27] proposed a distributed interference management technique for 2×2 *X* interference channel under a distributed manner. The two transmitters send the intended symbols to one desired receiver, where the interference signals are aligned based on the delayed CSIT knowledge. The distributed STIA technique has high transmitter energy consumption and a single receiver has to decode multiple intended symbols over the time slots, which will have significant impact on the decoding complexity at the receiver-end.

The proposed R-STIA transmission technique drastically decreases the interference misalignment for two intended receivers by the introduction of an additional receiver, efficient alignment and reconstruction of the interference signals. The transmitter energy consumption is subsequently reduced, since the proposed scheme activates only two transmitters over the time slots. The introduction of an additional (unintended) receiver aid in improving the desired signal strength drastically at the intended receiver-end. By considering the cooperation between the intended and unintended receivers, the decoding complexity is considerably reduced due to less interference misalignment at the intended receiver-end [28].

Although there are several other approaches for interference alignment, the proposed scheme has interesting methods for aligning the interference signals; the user 1 received signals from transmitters, $Y_{\left(1\right)}^{\left[1,\right]}$ and relay, $Y_{\left(1\right)}^{\left[R\right]}$ , are recovered and reconstructed at the unintended (additional) receiver during time slot 1. Using multiple antennas, the transmitters signals, { $Y_{\left(3\right)}^{\left[1\right]}$ , $Y_{\left(2\right)}^{\left[1\right]}$ } and the relay signals, $Y_{\left(3\right)}^{\left[R\right]}$ , $Y_{\left(2\right)}^{\left[R\right]}$ , are recovered at the unintended receiver-side, which assist in improving the receiver 1 signal strength drastically. Likewise, the received signals over time slot 2, { $Y_{\left(2\right)}^{\left[2\right]}$ , $Y_{\left(R\right)}^{\left[2\right]}$ } and time slot 3, { $Y_{\left(3\right)}^{\left[3\right]}$ , $Y_{\left(R\right)}^{\left[3\right]}$ }, can also be recovered, respectively, from the interference signals received through the additional receiver.

## 6. The K -User Relay-Aided MIMO Interference Channel

In this section, we characterize the K -user MIMO interference channel with a MIMO relay under limited CSIR knowledge. We restrict the number of K -user communication pairs to be lesser than the number of transmitter antennas, M, where K≤M .

**Time slot 1**: Let us consider K -time slots, where the transmitter sends M symbols via the relay to the corresponding M-antenna receiver; here, the K -user M×2 MIMO interference channel with a relay and an M-antenna receiver, has local CSIR knowledge. In the *k*th time slot, the (1≤k≤K) transmitter sends M desired symbols to the M-antenna receiver using the R-STIA technique; the symbols are $X^{\left[k\right]}\left[k\right]={\left[u_{1}^{\left(k\right)},u_{2}^{\left(k\right)},\ldots ,u_{M}^{\left(k\right)}\right]}^{T}$ ,

(32) $$\begin{array}{ccc} y_{j}^{\left(k\right)}\left(n\right) & = & \underset{\mathrm{desired}\;\mathrm{signal}\;+\;\mathrm{noise}}{\underset{︸}{h_{j,j}^{{\left[k,k\right]}^{T}}\left(n\right)u_{M}^{\left[k\right]}+Z_{j}^{\left[k\right]}\left(n\right)}}, \end{array}$$

where k∈{1,2,3,…,K}

(33) $$\begin{array}{ccc} y_{\left(j\right)}^{R}\left(n\right) & = & \underset{\mathrm{relay}\;\mathrm{signal}\;+\;\mathrm{noise}}{\underset{︸}{h_{j}^{\left[R,k\right]}\left(n\right)x_{j}^{k}\left(k\right)+Z_{j}^{\left[R\right]}\left(n\right)}}. \end{array}$$

During this time slot, the intended receiver has a weak interference and the unintended (additional) receiver has a strong interference; hence, the intended receiver has one equation, which contains M desirable symbols, whereas the unintended receiver has two equations containing M desirable symbols.

**Time slot 2**: The important objective of this time slot is to provide additional information to the unintended receiver, which assists in building (K−2) independent equations using the $u_{M}^{\left(k\right)}$ desired symbols. In this time slot, the receiver has CSIR knowledge for the current and past time periods because the relay forwards CSIR knowledge, such as the current and the outdated information to the receiver

(34) $$\begin{array}{c} \hat{X}{\;}^{\left[R\right]}\left[n\right]\;\;\;=\;\;\;\beta \left(V_{k}^{\left[1\right]}\left(n\right){\hat{u}}_{1}^{\left[k\right]}+V_{k}^{\left[1\right]}\left(n\right){\hat{u}}_{2}^{\left[k\right]}+,\ldots ,V_{k}^{\left[k\right]}\left(n\right){\hat{u}}_{M}^{\left[k\right]}\right), \end{array}$$

(35) $$\begin{array}{c} \left|\right|h_{\left[c_{k}\right]}^{{\left[K,k\right]}^{T}}\left(n\right){\left|\right|}_{2}{\bar{h}}_{\left[n-c_{k},⊥\right]}^{{\left[K,k\right]}^{T}}\;{\left(n\right)}^{T}\;\;\;=\;\;\beta h_{\left[c_{k}\right]}^{{\left[k,R\right]}^{T}}\;\left(n\right)V_{k}^{k}\;\left(n\right),\;k\in K. \end{array}$$

**Time slot n **: where n∈(K+1,K+2,…,K+M−1) and $\left|\right|h_{\left[c_{k}\right]}^{{\left[K,k\right]}^{T}}\left(n\right){\left|\right|}_{2}$ are the 2-norm of $h_{\left[c_{k}\right]}^{{\left[K,k\right]}^{T}}\left(n\right)$ . The transmitter and relay repeatedly transmit the same symbol, until the desired symbols are received in the intended receivers. During this transmission, the unintended receiver recovers the interference signals, enabling the reconstruction of the desired signal information for the intended receivers:

(36) $$\begin{array}{cc} y_{j}^{k}\left(n\right)= & h_{j,j}^{{\left[k,k\right]}^{T}}\left(n\right)x_{j}^{k}\left(n\right)+h_{j,j}^{{\left[k,R\right]}^{T}}\left(n\right)x_{j}^{R}\left(n\right),\\ & +\sum_{j=1,j\neq k}^{K}h_{j,i}^{{\left[k,j\right]}^{T}}\left(n\right)x_{i}^{j}\left(n\right)+Z_{j}^{\left[k\right]}\left(n\right). \end{array}$$

Considering that the transmitter sends K(K−1) linear combinations, $F^{\left[k,k\right]}\left[W_{k}\right]$ , of multiple data symbols to all the desired users, such as

(37) $$\begin{array}{cc} F^{\left[k,k\right]}\left[W_{k}\right] & =h_{\left[c_{1}\right]}^{\left[k,k\right]}\left[n\right]u_{1}^{\left[k\right]}+h_{\left[c_{2}\right]}^{\left[k,k\right]}\left[n\right]u_{2}^{\left[n\right]}\\ & +,\ldots ,\;\;+h_{\left[c_{k}\right]}^{\left[k,k\right]}\left[n\right]u_{M}^{\left[n\right]}, \end{array}$$

the unintended (additional) receiver assists in removing the interference and decodes the (K−1) data symbols reliably at the intended receiver with a very high SNR. Hence, the intended receivers can identify and remove the corresponding interference data symbols using local CSIR knowledge and the unintended-receiver information. The relay transmits the signal vector over the time slot n in the form of $\hat{X}{\;}^{\left[R\right]}\left[n\right]\;\;=\;\;\beta \left(V_{k}^{\left[k\right]}\left(n\right){\hat{u}}_{M}^{\left[k\right]}\right)$

(38) $$\begin{array}{c} y_{j}^{k}\left(n\right)=\beta h_{j,i}^{{\left[R,k\right]}^{T}}\left(n\right)\sum_{j=1}^{K}V_{k}^{\left[k\right]}\left(n\right){\hat{u}}_{M}^{\left[k\right]}+Z_{j}^{\left[R\right]}\left(n\right). \end{array}$$

It is important to design the beamforming matrices for aligning the interference signals from the transmitter and relay. To accomplish this goal, the beamforming matrices should satisfy the consecutive R-STIA conditions:

$$\begin{array}{c} \underset{K-1\times M}{\underset{︸}{\left[\begin{array}{c} h_{\left[c_{1}\right]}^{{\left[1,k\right]}^{T}}\left(k\right)\\ h_{\left[c_{2}\right]}^{{\left[\left(k-1\right),k\right]}^{T}}\left(k\right)\\ h_{\left[c_{3}\right]}^{{\left[\left(k+1\right),k\right]}^{T}}\left(k\right)\\ .\\ .\\ .\\ .\\ h_{\left[c_{k}\right]}^{{\left[K,k\right]}^{T}}\left(k\right) \end{array}\right]}}=\beta \underset{K-1\times M}{\underset{︸}{\left[\begin{array}{c} h_{\left[R_{1}\right]}^{{\left[1,R\right]}^{T}}\left(n\right)\\ h_{\left[R_{2}\right]}^{{\left[\left(k-1\right),R\right]}^{T}}\left(n\right)\\ h_{\left[R_{k}\right]}^{{\left[\left(k+1\right),R\right]}^{T}}\left(n\right)\\ .\\ .\\ .\\ .\\ h_{\left[R_{k}\right]}^{{\left[K,R\right]}^{T}}\left(n\right) \end{array}\right]}}\underset{M\times M}{\underset{︸}{V_{k}^{\left[k\right]}\left(n\right)}}. \end{array}$$

It is necessary to design the beamforming matrix, $V_{k}^{\left[k\right]}\left(n\right)$ , such that the intended receivers receive the same information during the time slots, until the data symbols are recovered. Where n represents time slots, n∈(1,2,…,k) ,

(39) $$\begin{array}{c} V_{k}^{\left[k\right]}\left(n\right)=\frac{1}{\beta }\left(\hat{H}{\left(n\right)}^{†}\left(\hat{H}\left(n\right)\hat{H}{\left(n\right)}^{†}\right)\hat{H}\left(n\right)\right). \end{array}$$

K -users transmit *M* data symbols, where the beamforming matrices are $V_{k}^{\left[k\right]}\left(n\right)=C^{M\times M}$ . To attain this objective, the beamforming matrices should satisfy the R-STIA condition $\hat{H}\left(n\right)=\beta \hat{H}\left(n\right)V_{k}^{\left[k\right]}\left(n\right)$ , where $\hat{H}\left(n\right)$ represents the channel matrices containing the desired channel coefficients, after applying $V_{k}^{\left[k\right]}\left(n\right)$ to the relay beamforming matrix. $\hat{H}\left(n\right)$ , is the channel matrix from the relay to all the receivers in the *n*th time slot. The complementary matrix, $H_{\left[c_{k}\right]}^{{\left[K,k\right]}^{T}}\left(n\right)$ , is denoted as follows:

(40) $$\begin{array}{c} H_{\left[c_{k}\right]}^{{\left[K,k\right]}^{T}}\left(n\right)=\left[h_{\left[c_{1}\right]}^{{\left[K,k\right]}^{T}}\left(n\right),h_{\left[c_{2}\right]}^{{\left[K,k\right]}^{T}}\left(n\right),\ldots ,h_{\left[c_{k}\right]}^{{\left[K,k\right]}^{T}}\left(n\right)\right]. \end{array}$$

Therefore, we can calculate the relationship between the input and output channel coefficients:

(41) $$\begin{array}{c} \left[\begin{array}{c} y_{\left[c_{1}\right]}^{\left[1\right]}\left(1\right)\\ y_{\left[c_{2}\right]}^{\left[(K+1)\right]}-\;\;\sum_{k=2}^{\left(K\right)}y_{\left[c_{k}\right]}^{\left[k\right]}\left(n\right)\;\;\\ y_{\left[c_{3}\right]}^{\left[(K+2)\right]}-\;\;\sum_{k=2}^{\left(K\right)}y_{\left[c_{k}\right]}^{\left[k\right]}\left(n\right)\;\;\\ .\\ .\\ .\\ .\\ y_{\left[c_{k}\right]}^{\left[(K+M-1)\right]}\;\;-\;\;\sum_{k=2}^{\left(K\right)}y_{\left[c_{k}\right]}^{\left[k\right]}\left(n\right)\;\; \end{array}\right]\;\;\;\;=\;\;\;\;\left[\begin{array}{c} h_{\left[c_{1}\right]}^{{\left[1,k\right]}^{T}}\left(1\right)\\ \left|\right|h_{\left[c_{2}\right]}^{{\left[\left(k-1\right),k\right]}^{T}}\left(n\right){\left|\right|}_{2}{\bar{h}}_{[c_{2}],⊥}^{{\left[\left(k-1\right),k\right]}^{T}}\left(n\right)\\ \left|\right|h_{\left[c_{3}\right]}^{{\left[\left(k+1\right),k\right]}^{T}}\left(n\right){\left|\right|}_{2}{\bar{h}}_{[c_{3}],⊥}^{{\left[\left(k+1\right),k\right]}^{T}}\left(n\right)\\ .\\ .\\ .\\ .\\ \left|\right|h_{\left[c_{k}\right]}^{{\left[K,k\right]}^{T}}\left(n\right){\left|\right|}_{2}{\bar{h}}_{[c_{k}],⊥}^{{\left[K,k\right]}^{T}}\left(n\right) \end{array}\right]\;u_{k}^{\left[k\right]}\left(n\right)\;\;\;+\;\;\;\left[\begin{array}{c} z_{\left[c_{1}\right]}^{\left[1\right]}\left(1\right)\\ {\hat{z}}_{\left[c_{2}\right]}^{\left[(K+1)\right]}-\;\;\sum_{k=2}^{\left(K\right)}z_{\left[c_{k}\right]}^{\left[k\right]}\left(n\right)\\ {\hat{z}}_{\left[c_{3}\right]}^{\left[(K+2)\right]}-\;\;\sum_{k=2}^{\left(K\right)}z_{\left[c_{k}\right]}^{\left[k\right]}\left(n\right)\\ .\\ .\\ .\\ .\\ {\hat{z}}_{\left[c_{k}\right]}^{\left[(K+M-1)\right]}-\;\;\sum_{k=2}^{\left(K\right)}z_{\left[c_{k}\right]}^{\left[k\right]}\left(n\right) \end{array}\right]\\ ⟹{\hat{y}}_{c_{1}}^{\left[1\right]}\left(1\right)=h_{\left[c_{1}\right]}^{{\left[1,1\right]}^{T}}\left(1\right)\left\{u_{\left[1\right]}^{1}+u_{\left[2\right]}^{1}\right\}+{\hat{z}}_{\left[c_{1}\right]}^{\left[1\right]}\left(1\right), \end{array}$$

where n={1,2,3,…,K}. We can conclude that interference-free communication is achievable without CSIT knowledge at the base station, provided the Alamouti space-time transmission technique and the MMSE precoder method are used for managing and aligning the interference signals using the delayed CSIR knowledge between the base stations and the mobile users.

## 7. Numerical Results

The proposed three-user MIMO interference channel model with a shared relay as shown in Figure 2. In this section, we consider K = 3 users, where two symbols are transmitted on each sub-carrier over the time slots. We also assume that the transmitters and relay each with two antennas (M=2) and a receiver equipped with single antenna (N=1) . The MMSE linear precoder with 256 quadrature amplitude modulation (QAM) is used in the simulation. The channel vector of each transmitter and relay is considered as an i.i.d complex Gaussian with zero mean and unit variance MIMO space-time-correlated Rayleigh fading channels. We also considered that the transmit power for each transmitter (P) and relay $\left(P_{R}\right)$ are 50 mW are fixed throughout the simulation.

**Figure 7:**
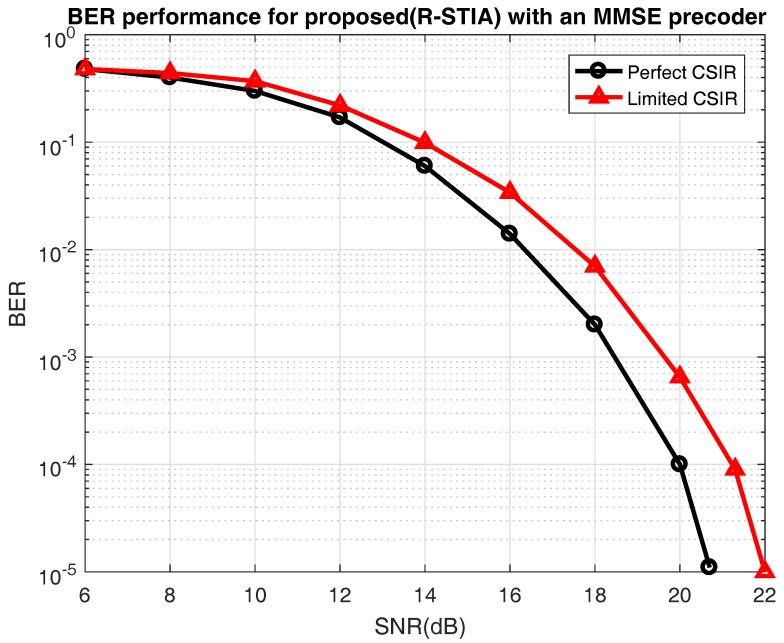
Bit error rate (BER) performance for proposed the relay space-time interference alignment (R-STIA) with perfect and limited channel state information at the relay (CSIR) knowledge.

In Figure 7, the BER performance is compared in two main scenarios: with limited CSIR knowledge and with perfect CSIR knowledge [18]. Thus, the proposed scheme relies on a limited CSIR knowledge because, over a time slot, only two transmitters are active; whereas, with a perfect CSIR, the relay knows all the channel state information. At BERs of $10^{-3}$ and $10^{-4}$ , in the perfect CSIR case the SNR increases from 20 dB to 21.3 dB respectively, whereas, in the proposed limited CSIR case, the SNR varies from 18.6 dB to 21.3 dB. The simulation result shows that the perfect CSIR case performance is 1.5 dB better than the proposed limited CSIR case. This result is predictable, since the relay has only limited channel state information over the time slots.

**Figure 8:**
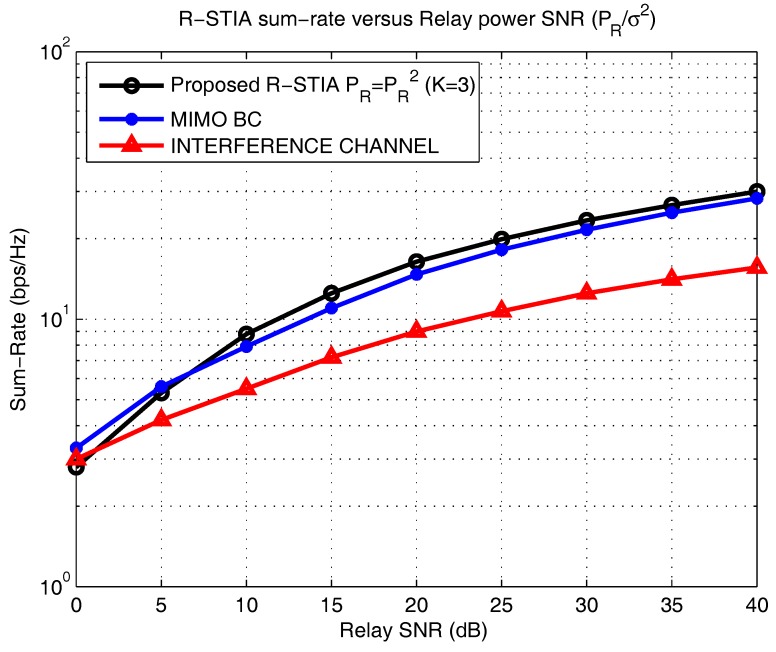
Sum-rate for the proposed relay space-time interference alignment (R-STIA) technique.

Figure 8 displays the power allocation strategy versus the computed achievable sum-rate [17,22]. We mainly focus on three major schemes; in the proposed R-STIA technique, the sum-rate is computed for K = 3 users, under an average power constraint. The R-STIA technique is compared with the MIMO broadcast channel and the interference channel schemes, where at SNRs of 20 dB and 40 dB, the sum-rate increases from 17.5 bps/Hz to 30 bps/Hz, respectively; the MIMO broadcast channel achieves an increase from 15.7 bps/Hz to 27.4 bps/Hz and the interference channel scheme from 8.5 bps/Hz to 15.5 bps/Hz. This drastic variation in the sum-rate is predicted because, in the proposed scheme, the additional receiver and limited CSIR information enable an increase in the sum-rate compared to the MIMO broadcast and interference channel schemes.

**Figure 9:**
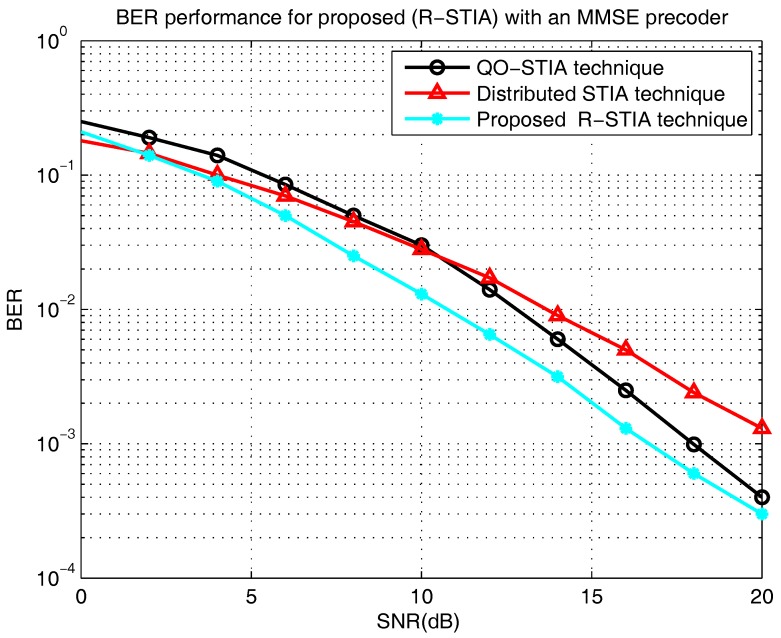
Bit error rate (BER) performance of the proposed relay space-time interference alignment (R-STIA) with an minimum mean square error (MMSE) precoder.

Figure 9 presents the bit error rate versus the SNR (dB) [18]. The proposed R-STIA technique is compared with the QO-STIA and the distributed R-STBC techniques. At BERs of $10^{-2}$ and $10^{-3}$ , in the proposed technique, the SNR increases from 10.8 dB to 16.3 dB, respectively, whereas the relay QO-STIA technique achieves an SNR increase from 2.5 dB to 18 dB and the distributed STIA technique from 14 dB to 21 dB. This extreme variation is predictable because the proposed techniques relies on the MMSE linear precoder, which, in turn, helps to improve the BER performance.

**Figure 10:**
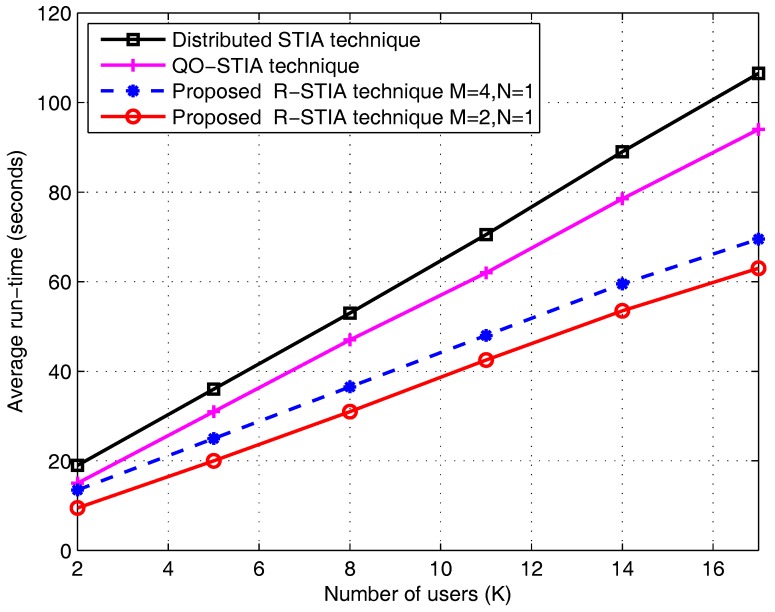
Average run-time versus the number of users.

In Figure 10, we plot the average run-time (seconds) versus the number of users ( K ). The proposed R-STIA technique average run-time (seconds) is compared with the distributed STIA and QO-STIA techniques. At K = 16 users case, the proposed R-STIA technique for M=2 , N=1 antenna settings average run-time is 60 s. However, the QO-STIA and distributed STIA techniques average run-time is 90 s and 100 s, respectively. The simulation results show that the proposed R-STIA technique enhances the average run-time performance (seconds) compared to the distributed STIA and QO-STIA techniques. This result is predictable, since the proposed R-STIA technique reduces the decoding complexity due to the presence of additional receiver and less interference misalignment at the intended receiver-end. Moreover, the cooperation between the intended and unintended receivers also helps to reduce the decoding complexity of the proposed scheme.

## 8. Conclusions

This paper has addressed the performance of the IA transmission for a three-user M×2 MIMO relay-aided broadcast channel with limited CSIR knowledge. We have proposed an alternative transmission named the R-STIA technique, in which the unintended receiver incorporates two unknown receiver signals from the two active transmitters. The proposed R-STIA technique recovers and reconstructs the transmitter and relay signals at the unintended receiver-end by considering the cooperation between the receivers. In addition, the Alamouti space-time transmission and an MMSE linear precoder were employed at the transmitters and relay in order to detect the presence of interference signals at the unintended receiver. Through the numerical results, it was confirmed that the proposed R-STIA technique achieves a better sum-rate and BER performance compared to the existing broadcast channel schemes. Furthermore, the Alamouti space-time transmission and the MMSE linear precoder ensures the better alignment of the interference signals at the receiver-end.

## Abbreviations

The following abbreviations are used in this manuscript:

R-STIA	Relay space-time interference alignment
MIMO	Multiple-input-multiple-output
MISO	Multiple-input-single-output
CSIR	Channel state information at the relay
CSIT	Channel state information at the transmitter
STIA	Space-time interference alignment
CSI	Channel state information
SNR	Signal-to-noise ratio
BC	Broadcast channel
BS	Base stations
BER	Bit error rate
DoF	Degree of freedom
i.i.d	Independent and identically distributed
MMSE	Minimum mean square error
ZL-LP	Zero forcing-linear precoding method
TIM-MP	Topology interference management performance with a message passing
PIMAC	Point-to-point interfering with multiple access relay channel
QO-STIA	Quasi-orthogonal space-time interference alignment
